# Role of biological markers in the clinical outcome of colon cancer

**DOI:** 10.1038/sj.bjc.6600569

**Published:** 2002-10-07

**Authors:** O Nanni, A Volpi, G L Frassineti, F De Paola, A M Granato, A Dubini, W Zoli, E Scarpi, D Turci, G Oliverio, A Gambi, D Amadori

**Affiliations:** Istituto Oncologico Romagnolo, Corso Mazzini 65, 47100 Forlì, Italy; Department of Medical Oncology, Pierantoni Hospital, Via Forlanini 34, 47100 Forlì, Italy; Pathology Unit, Pierantoni Hospital, Via Forlanini 34, 47100 Forlì, Italy; Department of Oncology, Santa Maria delle Croci Hospital, via Missiroli 10, 48100 Ravenna, Italy; Department of Oncology, Infermi Hospital, via Settembrini 2, 47900 Rimini, Italy; Department of Oncology, Degli Infermi Hospital, viale Stradone 9, 48018 Faenza, Italy

**Keywords:** colon cancer, clinical outcome, biofunctional features, 5-FU adjuvant chemotherapy

## Abstract

We investigated a number of biological markers, evaluated under strict intralaboratory quality control conditions, in terms of their role in predicting clinical outcome of patients with colon cancer treated with 5-FU-containing regimens. Colon cancer tissue from 263 patients enrolled onto two randomised clinical trials were studied for their cytofluorimetrically determined DNA content and their immunohistochemically evaluated microvessel density, vascular endothelial growth factor expression, thymidylate synthase expression and tumour lymphocyte infiltration. Disease-free survival and overall survival of patients were analysed as a function of the different variables. At a median follow up of 57 months, age, gender and Dukes' stage showed an impact on disease-free survival, whereas no biological marker emerged as an indicator of better or worse disease-free survival. Only histological grade and Dukes' stage were found to influence overall survival. The different biological variables, studied with particular attention for determination reliability, proved to have no impact on the clinical outcome of patients with colon cancer. Therefore, other markers must be identified to complement clinico-pathological variables in the management of this disease.

*British Journal of Cancer* (2002) **87**, 868–875. doi:10.1038/sj.bjc.6600569 www.bjcancer.com

© 2002 Cancer Research UK

## 

Clinical experiences have repeatedly shown that colon cancers of the same histotype or at the same pathological stage have a different clinical outcome and may respond differently to chemotherapy. In the last 20 years researchers have tried to identify biological markers capable of predicting clinical outcome and tumour sensitivity or resistance to cytotoxic drugs.

The first studies focused mainly on cellular features such as cell proliferation and DNA ploidy, but since then genetic alterations (p53, K-ras, deletions involving chromosome 18q, microsatellite instability), enzymatic activities (thymidylate synthase (TS)), dihydropyrimidine dehydrogenase, thymidine phosphorylase, neoangiogenesis-related markers (vascular endothelial growth factor (VEGF), microvessel density (MVD)) and lymphocyte infiltration have been investigated.

Controversial results have been obtained and almost all these variables have been classed as category IIB (well studied but not sufficient for category I or IIA) or category III (not yet studied sufficiently to meet criteria for category I, IIA or IIB) by the American Joint Committee on Cancer Prognostic Factors Consensus Conference (Compton *et al*, 2000). The main limitations of the studies are the small sample size and heterogeneity of case series. Furthermore, only specific groups of markers have been investigated in individual studies.

In the present study a number of biological markers, larger than any previously investigated and selected among those considered as the most promising for their potential role as prognostic variable or indicator of response to chemotherapeutic regimens, were determined in parallel on each tumour.

In particular, in a cohort of 330 patients enrolled onto two randomised studies and given 5-FU-containing chemotherapeutic associations with different modulators (levamisole, folinic acid, methotrexate), MVD, VEGF expression, cytofluorimetrically-determined nuclear DNA content (ploidy), tumour lymphocyte infiltration and TS expression were analysed together with conventional clinico-pathological variables. We decided not to include p53 expression, which for some time has been the object of considerable research, in our biologic characterisation as our own experience and that of other authors did not leave us confident about the reliability of results obtainable from samples histologically fixed for uncontrolled and variable times (Daidone *et al*, 1998).

The series of patients was recruited by one of five cooperative groups taking part in two multicenter randomised clinical protocols (Di Costanzo *et al*, 1999).

Given the contradictory results on the clinical relevance of biological markers, special attention was paid to determination reproducibility through strict intralaboratory quality controls.

## MATERIALS AND METHODS

### Patients

From March 1992 to December 1998 five Italian cooperative groups conducted two large national multicentre randomised clinical trials on adjuvant chemotherapy in colon cancer. In both studies eligibility criteria were as follows: histologically proven and radically resected primary intraperitoneal colon adenocarcinoma (Dukes' Stage B2, B3, or C) and ECOG Performance Status <2. The definition of colon cancer included any lesion of the large bowel that did not require the opening of the pelvic peritoneum to define the distal extent of the tumour and/or any lesion localised at 12 cm from the pectinate line. The inferior margin of the tumour had to be above the peritoneal reflection. The absence of metastases had to be ascertained before randomisation by liver ultrasonography or CT scan, chest X-ray and exploratory laparatomy during surgery.

The first trial, INTACC 01 (1703 patients), was concluded in February 1995. Patients were randomised as follows: arm A, 5-fluorouracil (5-FU) 450 mg m^−2^ i.v. bolus on days 1–5+levamisole (LEVA) 150 mg per os on days 1–3; arm B, 5-FU 370 mg m^−2^ i.v. bolus on days 1–5 preceded by an i.v. bolus of 6-S-leucovorin (6-S-LV) 100 mg m^−2^ on days 1–5+LEVA given as in arm A. In both arms, 5-FU courses were repeated at 4-week intervals for six cycles and LEVA was given every other week for a total of 24 weeks (Di Costanzo *et al*, 1999)

INTACC 02, activated in February 1995 and concluded in December 1998, recruited 1945 patients. Patients were randomised as follows: arm A, as arm B of INTACC 01; arm B, methotrexate (MTX) 40 mg m^−2^ i.v. bolus on days 1 and 8, 5-FU 600 mg m^−2^ i.v. bolus on days 2 and 9, LEVA 150 mg per os on days 1–3. In both arms, 5-FU courses were repeated at 4-week intervals for six cycles and LEVA was given every other week for a total of 24 weeks (Lionetto, 1999).

For both studies, clinical data were collected at the time of randomisation, which was carried out within 60 days of surgery. The study was examined and approved by the Ethics Committee of the Local Health and Social Services of each centre in accordance with the ethical standards laid down in the 1964 Declaration of Helsinki. All patients gave oral or written informed consent.

Randomisation procedures were carried out within individual cooperative groups. Timing and dose modification were planned on the basis of toxicity registered.

A clinical, haematological, instrumental (chest X-ray, and liver ultrasonography or CT scan) and biochemical assessment for each patient was performed at 6-month intervals for the first 2 years, after which all examinations were carried out once a year. In addition, following randomisation, all patients underwent an annual barium enema and/or colonscopy.

A total of 330 patients were recruited from March 1992 to December 1998 by *Istituto Oncologico Romagnolo*, one of the five Italian cooperative groups participating in INTACC 01 and INTACC 02.

### *In vitro* determinations

Paraffin-embedded histological blocks were available for 263 of the 330 randomised cases.

On the basis of the priority of biological determinations, the amount of available tumour material and determination feasibility, information on TS was available for 257 patients, on lymphocyte infiltration for 256, on MVD for 251, on ploidy for 243 and on VEGF for 187. All biological determinations were carried out in only one laboratory (Forlì), which participates, for ploidy, or is the coordinating center, for MVD, in national Quality Control Programmes. For lymphocyte infiltration, VEGF and TS, the reliability and reproducibility of results were guaranteed by blind determination performed by at least two observers.

### Immunohistochemical determinations

Tumour samples were fixed in 10% formalin. Four-micrometre sections were mounted on positive-charged slides (Bio-Optica, Milan, Italy), deparaffinised with xylene, rehydrated and endogenous peroxidase activity was blocked by 3% hydrogen peroxide solution. After adequate antigen retrieval, the sections were treated for non-specific binding with 3% bovine serum albumin in PBS for 20 min and then incubated for 1 h at room temperature with primary antibodies. The sections were washed with PBS and incubated with biotinylated anti-mouse secondary antibody. After rinsing with PBS, sections were incubated with streptavidin-peroxidase conjugate (Dako Corporation, LSAB+ kit, Carpinteria, CA, USA). Sections were then rinsed in PBS, and antibody binding was detected by staining with diaminobenzidine/hydrogen peroxidase chromogen solution (DAB+, liquid substrate-chromogen solution, Dako Corporation). Sections were rinsed in deionised water, cell nuclei were counterstained blue by Mayer's Haemalum and the sections were mounted in Eukitt (Bio-Optica).

For microvessel density determination, sections were incubated with polyclonal antibody against human Factor VIII-related antigen (Dako Corporation) at a 1 : 300 dilution in PBS containing 10% goat serum for 30 min at room temperature. After washing in PBS, sections were incubated with an antirabbit biotinylated antibody and positivity detected by an immunoperoxidase reaction (Kit Supersensitive, Biogenex, San Ramon, CA, USA) for 20 min. Blood vessels within the tissue were used as a positive internal control for Factor VIII positivity. Each set of samples contained a negative control in which primary antibody was omitted.

MVD, evaluated by scoring contiguous fields, was expressed according to Weidner's method as the number of microvessels mm^−2^ (Medri *et al*, 2000). The scoring and count were performed blindly by two observers. The laboratory (Forlì) where MVD was determined is the Reference Center for a National Quality Control Programme sponsored by the Italian Ministry of Health.

Thymidylate Synthase expression was determined using a monoclonal antibody (clone 106, Neomarker, Union City, CA, USA) that specifically reacts with TS protein in formalin-fixed, paraffin-embedded human tissue, at a dilution of 1 : 80 in PBS. TS antigen retrieval was performed by microwaving the slides at 600 W for 18 min in 10 mM citrate buffer (pH 6.0) followed by cooling at room temperature for at least 20 min. Control tumour slides (LoVo and WiDr tumour cell lines) were stained in parallel with the antibody. Immune serum was omitted in negative controls.

Vascular endothelial growth factor expression was determined using a pre-diluted polyclonal antibody that specifically reacts with VEGF isoforms 121, 165, 189, and 206 in formalin-fixed, paraffin-embedded human tissue (Biogenex, San Ramon, CA, USA). VEGF antigen retrieval was performed by microwaving the slides at 750 W for 15 min in 10 mM citrate buffer (pH 6.0) followed by cooling at room temperature for at least 20 min. Control tumour slides (HL-60 and K562 tumour cell lines) were stained in parallel with the antibody. Immune serum was omitted in negative controls.

For both VEGF and TS determinations, each section was scored by two separate observers (FDP, AMG) at light-microscope (200× and 400×) and, in cases when the difference exceeded 10%, by a third observer. Immunoreactivity was expressed as the ratio between the percentage of immunopositive areas in relation to the whole section of invasive neoplastic tissue. The expression was also quantitated using a grading system based on intensity of staining (0–3).

Tumor lymphocyte infiltration was evaluated semi-quantitatively as absent, poor, moderate or marked (0 to 3).

Flow cytometric analysis was performed on cell suspensions obtained from 50-μm paraffin-embedded sections using a modified version of the procedure developed by Hedley *et al* (1983). Briefly, tissue was minced, digested in 1% pepsin solution, pH 1.5, for 30 min at 37°C. Cells were then resuspended in RPMI, filtered through a disposable 40 μm filter assembly (RATCOM) and disgregated nuclei were incubated with 500 μl of Bauer solution, pH 7.2 (RNAse) (0.2 Ku ml^−1^), PEG 8000 (30 mg ml^−1^), tri-Sodium citrate (1.2 mg ml^−1^), Triton X-100 (100 μg ml^−1^) and propidium iodide (2.5 μg ml^−1^)) for 20 min at 37°C.

Nuclei were stained with 500 μl of a solution containing propidium iodide (25 μg ml^−1^), PEG 8000 (300 mg ml^−1^), Triton X-100 (100 μg ml^−1^) and NaCl (9.4 mg ml^−1^). The samples were stored for 30–60 min before flow cytometric analysis.

All samples were analysed using FACS Vantage flow cytometer (Becton Dickinson; San Jose, CA, USA) equipped with a water-cooled argon-ion laser. Data acquisition was performed using CELLQuest software and for each sample at least 30 000 events were collected and stored for subsequent analysis. Data were elaborated using Modfit (DNA Modelling System) software and expressed as fractions of cells in the different cycle phases. A histogram was considered interpretable if the coefficient of variation of the G_0_/G_1_ peak was less than 5%. DNA ploidy was defined as DNA Index (DI) and tumours with a DI≠1 were considered to be aneuploid.

### Statistical analysis

The relationship between VEGF, TS, MVD and clinico-pathological or biological characteristics was analysed using a non-parametric ranking statistic (Median test), and the Spearman's correlation coefficient (r_s_) was used to investigate the relation between the different biological markers considered as continuous variables.

DFS was calculated as the period from the date of surgery until the first documented evidence of new disease manifestation in locoregional or distant sites, death due to any cause or occurrence of a new non-colorectal malignancy. For OS, death due to any cause was considered as an event. Four-year DFS and OS and the 95% Confidence Interval (95%CI) were obtained using Kaplan-Meier estimates (Kaplan, Meier, 1958). The median follow up of the entire cases series of 263 patients entered onto the biologic characterisation study was 57 months (range 2–104).

The possible role of the quantitative markers (VEGF, TS, MVD) in influencing clinical outcome was studied by analysing each of them as continuous and dichotomous variables. Median values were used as cut-off to identify groups with different risk of relapse or death.

The null hypothesis concerning the different impact of each dichotomized biologic marker on clinical outcome was tested in univariate analysis by a stratified logrank test, according to treatment schedule. This stratification allowed to adjust for possible confounding effects of treatment on clinical outcome, although it must be pointed out that all treatment combinations contained 5-FU and differed only in the modulating compound (LEVA, 6-S-LV, Methotrexate). Furthermore, the clinical results from INTACC 01 did not highlight any difference between the two arms. The impact of each biological marker on clinical outcome was also analysed in different Cox regression models, stratified by treatment schedule and containing age, gender, Dukes' stage and histological grade.

All *P* values were based on two-sided testing and statistical analyses were carried out with SAS Statistical Software (SAS Institute Inc., 1989).

## RESULTS

The clinical, pathological and biological features of the 263 patients for whom paraffin-embedded archive material was available for biological characterisation are reported in Table 1

**Table 1:**
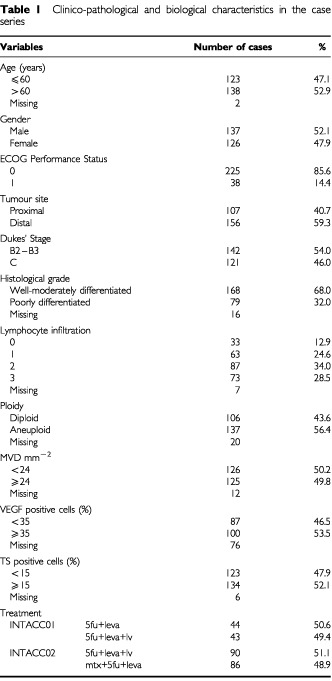
Clinico-pathological and biological characteristics in the case series

.

Median age was 60 years (range 28–79) and males and females, as well as patients with Dukes' Stage B or C tumours, were evenly distributed in the case series. About 60% of tumours were localised in the distal tract of the colon and more than two-thirds of lesions were well or moderately differentiated. More than 80% of patients had a good Performance Status (ECOG=0). Of the 263 patients, 87 patients were enrolled onto INTACC 01 and the remaining 176 onto INTACC 02. Patients were very well-balanced in the two arms of each study.

With regard to the biofunctional characterisation, differing degrees of tumour lymphocyte infiltration were observed in about 87% of cases. The frequency of diploid tumours at flow cytometric analysis was 44% and, among the 56% of aneuploid tumours, 20% showed multiple cell clones (data not shown).

MVD ranged from 4 to 95 microvessels mm^−2^ with a median value of 24 and VEGF-expressing cells varied from 0 to 100%, with a median value of 35%. The fraction of TS-expressing cells ranged from 0 to 90% in the different tumours, with a median value of 15%.

Immunostaining intensity was evaluated for both VEGF and TS. VEGF staining was not observed in 6% of cases, whereas staining intensities 1, 2 and 3 were found in 34, 44 and 16% of tumours, respectively. TS immunostaining was not present in 27% of cases and intensities 1, 2 and 3 were observed in 28, 34 and 11% of cases, respectively.

**Figure 1:**
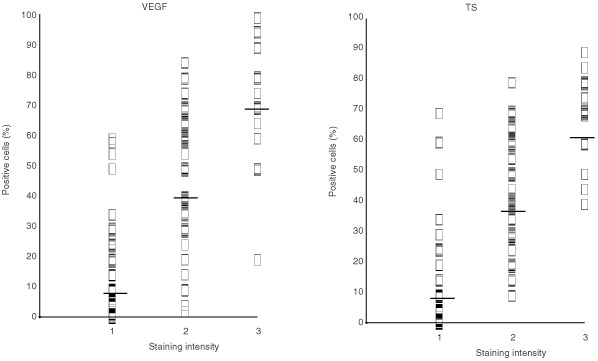
Relationship between staining intensity and percentage of VEGF and TS immunopositive cells.

A highly significant direct relation between the percentage of immunopositive cells and staining intensity (Figure 1

) was observed for both VEGF and TS. For this reason, only the percentage of immunopositive cells was considered in the subsequent analyses.

The analysis of age, MVD, TS and VEGF, considered as continuous variables, showed no correlation between any of the markers, with the exception of a significant direct correlation between TS and age, albeit with a very poor coefficient (r_s_=0.16) (data not shown).

The evaluation of the biologic markers in the different subgroups defined according to clinico-pathological characteristics showed that MVD median value was significantly higher in patients with an ECOG performance status of 1 than of 0, in proximal tumours and, unexpectedly, in those with well-moderately rather than poorly differentiated lesions. A higher median value of VEGF-expressing cells was observed in males than in females. TS median value was significantly higher in older than in younger patients and in well-moderately with respect to poorly differentiated lesions. Moreover, TS expression was significantly related to lymphocyte infiltration, with a median value 10 times higher in the absence or presence of a weak compared to massive infiltration. No relation was observed between ploidy and MVD, VEGF or TS (Table 2

**Table 2:**
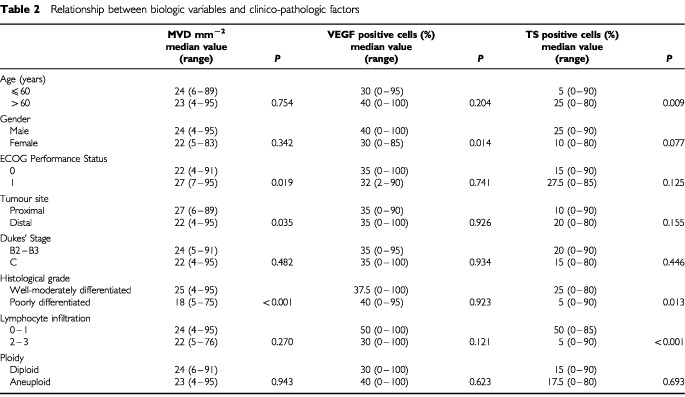
Relationship between biologic variables and clinico-pathologic factors

).

DFS and OS were analysed as a function of the different variables in univariate analysis (Table 3

**Table 3:**
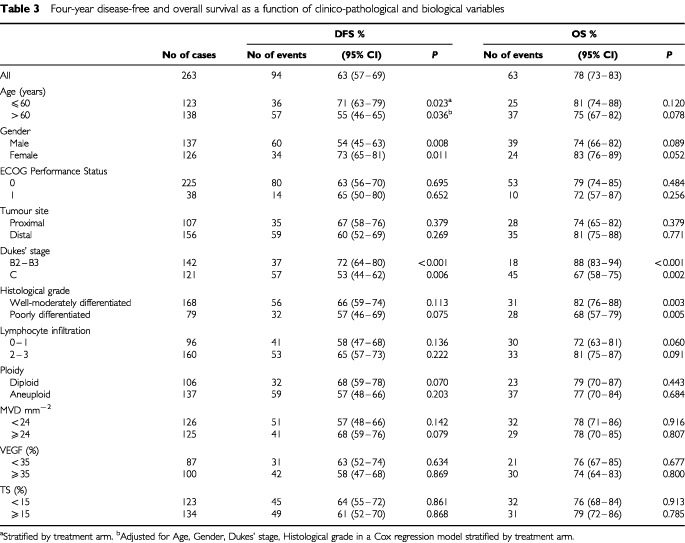
Four-year disease-free and overall survival as a function of clinico-pathological and biological variables

). Age, gender and Dukes' stage showed an impact on DFS, as expected and repeatedly reported in the literature (Sant, Eurocare Working Group, 1999; Skibber *et al*, 2001) with a significantly better prognosis for younger and female patients and for those with Dukes' stage B tumours. Only a slightly better DFS was observed for patients with well-moderately differentiated or highly vascularized tumours analysed in a Cox regression model and for those with diploid tumours, whereas no other biological marker was indicative of DFS.

With regard to OS, the relevance of age and gender was weakened, whereas it was confirmed for histological grade and Dukes' stage. Among the biological variables tested, a trend of better prognosis was observed for high-massive compared to low lymphocyte infiltration.

When VEGF, MVD, TS were analysed as continuous variables, the coefficient estimate for each unit increase was not different from 0 or, from a statistical point of view, did not highlight a role played by any variable on clinical outcome.

## DISCUSSION

Over the last 20 years clinical researchers have tried to identify biological markers that predict tumour sensitivity or resistance to clinical therapies and probability of recurrence. Ultimately, their usefulness in clinical decision-making depends on easy determination at the lowest possible costs. In colorectal cancer, for example, some newly proposed and investigated molecular markers, such as microsatellite instability (Elsaleh *et al*, 2000; Watanabe *et al*, 2001), appear promising, but not all laboratories are equipped for their determination. For this reason we felt it would be interesting to investigate a number of easily assessable biofunctional features of colorectal cancer, paying special attention to the reliability and reproducibility of determinations.

In our case series the frequency of aneuploid tumours was in line with the highest values reported in the literature, thus indicating that no aneuploid populations were lost during methodological manipulations, notwithstanding the use of paraffin-embedded material (Silvestrini *et al*, 1993; Flyger *et al*, 1999; Salud *et al*, 1999).

Literature data on the clinical relevance of ploidy are contradictory, showing a statistically significant role, a weak or non independent role or no prognostic relevance on clinical outcome (Silvestrini *et al*, 1993; Ratto *et al*, 1998; Flyger *et al*, 1999; Salud *et al*, 1999; Purdie and Piris, 2000; Skibber, *et al*, 2001). These controversial results can be attributed to heterogeneity of case series in terms of stage and treatment and may also be due to the length of follow-up. In our case series only a trend of better DFS, which was not observed for OS, was seen for patients with diploid tumours.

With regard to the neoangiogenesis process, it has been hypothesised that, in both experimental and clinical models, the increase in microvessel density, by favouring drug access, could be considered as a predictor of response to chemotherapy and can therefore be associated with a better prognosis. However, there are no definitive conclusions on the clinical relevance of MVD in patients with operable colon cancer treated with antimetabolites in an adjuvant setting. It is difficult to compare our findings with those reported in other studies due to the heterogeneity of case series with respect to stage and treatment. While some studies (Bossi *et al*, 1995; Frank *et al*, 1995; Mooteri *et al*, 1996; Takebayashi *et al*, 1996; Amaya *et al*, 1997; Takahashi *et al*, 1997; Banner *et al*, 1998; Giatromanolaki *et al*, 1999; Vermeulen *et al*, 1999) found a direct or no relation between high angiogenic activity and poorer prognosis, we observed a trend of a better prognosis for patients with high MVD tumours, in agreement with recently published results obtained on large case series (Lindmark *et al*, 1996; Abdalla *et al*, 1999). Similar results have already been reported in breast cancer (Protopapa *et al*, 1993; Axelsson *et al*, 1995; Medri *et al*, 2000). Whether this finding can be attributed to a lower clinical aggressiveness or to a higher chemosensitivity of high MVD tumours remains to be ascertained, although the latter hypothesis appears the most likely.

With regard to VEGF expression (Berney *et al*, 1998; Ishigami *et al*, 1998; Tokunaga *et al*, 1998; Cascinu *et al*, 2000; Lee *et al*, 2000; Maeda *et al*, 2000), no differences were observed between DFS and OS in patients with high or low VEGF-expressing tumours. It is also worthy of note that, differently from other studies (Amaya *et al*, 1997; Takahashi *et al*, 1997), we did not find any relation between MVD and VEGF expression. In our opinion the two markers are not necessarily linked to each other. The formation of new microvessels, and therefore MVD, may be the result of many factors such as tumour size, stage, presence of necrosis or ulceration, or levels of angiogenic peptides. Among these, VEGF is certainly one of the growth factors involved in initiating and stimulating the formation of intratumoral microvessels, but other factors may also play a important role.

Few papers have investigated the role of lymphocyte infiltration (Carlon *et al*, 1984; Svennevig *et al*, 1984; Jass, 1986; Harrison *et al*, 1994). In our study, no association was found between the presence of lymphocyte infiltration and clinical outcome, suggesting that the immunological response of the host to the primary tumour may not be related to metastatic potential, which is known to be the result of selected cell clones that have acquired the ability to deceive or overcome the host's immune strategies.

TS expression has been extensively studied in the last few years both as a prognostic factor and as a predictor of response to chemotherapy. Overall, studies have dealt with the relation between TS expression and clinical outcome of patients in both adjuvant and advanced settings. In metastatic disease, low TS levels have consistently been found to be predictive of responsiveness to 5-FU-based chemotherapy (Peters *et al*, 1994; Johnston *et al*, 1995; Kornmann *et al*, 1997; Leichman *et al*, 1997, Leichman, 1998; Lenz *et al*, 1998; Aschele *et al*, 1999; Bathe *et al*, 1999; Cascinu *et al*, 1999; Paradiso *et al*, 2000; Allegra, 2002). The situation in the adjuvant setting is less clear (Johnston *et al*, 1994; Findlay *et al*, 1997; Yamachika *et al*, 1998; Sanguedolce *et al*, 1998; van Triest *et al*, 2000; Takenoue *et al*, 2000; Edler *et al*, 2000; Cascinu *et al*, 2001; Wong *et al*, 2001; Tomiak *et al*, 2001; Allegra *et al*, 2002). With respect to the real prognostic impact of TS on the natural history of colorectal cancer, the few studies conducted on mainly small series of patients not treated with systemic therapy (Yamachika *et al*, 1998; Sanguedolce *et al*, 1998; van Triest *et al*, 2000; Takenoue *et al*, 2000; Edler *et al*, 2000, 2002) have shown inconclusive results.

In colorectal cancer patients treated with adjuvant 5-FU-containing chemotherapy, both high (Johnston *et al*, 1994; Takenoue *et al*, 2000; Edler *et al*, 2002) and low (Cascinu *et al*, 2001; Wong *et al*, 2001) TS expression were found to be associated with a better clinical outcome. Our results and those of other studies (Findlay *et al*, 1997; Yamachika *et al*, 1998; Tomiak *et al*, 2001) did not highlight that TS was capable of predicting clinical outcome.

In conclusion, our findings, obtained under strict quality control conditions for biological determinations and with close patient follow-up, indicate that MVD, VEGF and TS expression are not ready for ‘prime time’ and that other markers must be identified for a rational management of colon cancer patients.

On the basis of available literature data and in the light of the results from the present study, it is not possible to draw any definitive conclusions about the role of the investigated biomarkers in predicting the clinical outcome of patients with colorectal cancer. Even though it is not a diffuse custom among clinical researchers, future investigations should be carefully planned to achieve an adequate level of evidence that will allow the questions put forward to be answered.
